# Simulation of multibody systems with servo constraints through optimal control

**DOI:** 10.1007/s11044-016-9558-z

**Published:** 2016-11-10

**Authors:** R. Altmann, J. Heiland

**Affiliations:** 1 Institut für Mathematik MA4-5, Technische Universität Berlin, Straße des 17. Juni 136, 10623 Berlin, Germany; 2 Max Planck Institute for Dynamics of Complex Technical Systems, Sandtorstraße 1, 39106 Magdeburg, Germany

**Keywords:** Servo constraints, Inverse dynamics, High-index DAEs, Optimal control, Underactuated mechanical systems

## Abstract

We consider mechanical systems where the dynamics are partially constrained to prescribed trajectories. An example for such a system is a building crane with a load and the requirement that the load moves on a certain path.

Enforcing this condition directly in form of a servo constraint leads to differential-algebraic equations (DAEs) of arbitrarily high index. Typically, the model equations are of index 5, which already poses high regularity conditions. If we relax the servo constraints and consider the system from an optimal control point of view, the strong regularity conditions vanish, and the solution can be obtained by standard techniques.

By means of the well-known $n$-car example and an overhead crane, the theoretical and expected numerical difficulties of the direct DAE and the alternative modeling approach are illustrated. We show how the formulation of the problem in an optimal control context works and address the solvability of the optimal control system. We discuss that the problematic DAE behavior is still inherent in the optimal control system and show how its evidences depend on the regularization parameters of the optimization.

## Introduction

We consider mechanical systems with servo constraints; see, e.g., [7, 8, 19], for which a part of the motion is specified. This includes crane models where one seeks for an input that makes an end effector follow a prescribed trajectory. Often, these configurations are called *inverse dynamics* problems or, since the number of degrees of freedom exceeds the number of controls, *underactuated mechanical systems*. A recent overview of the diversity of given applications can be found, e.g., in [9, 27]. In this paper, we consider a two-car example [8, Sect. II], its generalization to $n$ connected cars, and an overhead crane example [5, Ex. 4]. In all of these examples, the model equations are *differentially flat*, which means that the input can be expressed and determined solely by means of the desired output and its derivatives [12].

In the direct modeling approach, the inputs are regarded as variables, whereas the desired output is formulated as a constraint. This constraint makes the model equations a system of differential-algebraic equations (DAE) even though the dynamics of the system may be given in the form of an ODE. Even more, the resulting DAE is typically of high index so that for its application in simulations, we need to employ a suitable *index reduction* [1, 5, 7]. Apart from the numerical difficulties that come with high index DAEs, an immediate drawback of the DAE approach is that a solution to the problem can only exist if the target trajectory is sufficiently smooth.

As a cure to both mentioned shortcomings of the DAE approach, we consider an optimal control approach that relaxes the constraints and balances the approximation to the target with the control effort. The relaxation of the constraint $Cx = y$ softens the strong regularity assumptions in the DAE setting. In theory, the desired trajectory $y$ may then be even discontinuous.

The same approach was discussed in [26], however, without analyzing the optimality system such that, due to otherwise inconsistent boundary conditions, it becomes necessary to introduce an additional regularization.

Apart from inconsistent boundary conditions, also nonsmooth data may cause problems—not in theory but in the practical application of the optimization approach. In other words, the DAE problematic is not simply gone since the weak coupling of the masses and the noncollocated sensors and activators may lead to oscillations in the output and strong peaks in the (unknown) input. A remedy is the penalization of the derivatives of the control force at the expense of a worse performance and of less standard systems of equations for the numerical realization.

In this paper, we analyze the optimal control approach and investigate methods for the solution of the resulting equation systems. In particular, the following issues are addressed:

1. The very weak coupling of input and output that, in the (DAE) limit, may lead to singular actuations. We will investigate the dependency of the penalty or regularization parameters and the behavior in the limit case.
2. Necessary and sufficient optimality conditions with an emphasis on their use for the solution of the optimization problem. Particularly, in the case of holonomic constraints, the formally derived first-order optimality conditions are preferable over alternative formulations, but they may not be solvable due to inconsistent data.

The paper is organized as follows. In Sect. 2, we give the formulation of the servo-constraint problem as a DAE for which additional holonomic constraints are allowed. The counterpart is then presented in Sect. 3 in which the same configuration is modeled as an optimal control problem. Here we formulate the optimality conditions, discuss the consistency conditions of the boundary data, and prove the existence of an optimal solution. The relation of the two approaches is then the topic of Sect. 4, in particular, the optimal control problem in which the input is not penalized is analyzed. Section 5 gives an overview of the different solution strategies. This includes the DAE case and the optimal control approach for which boundary-value problems have to be solved. In Sect. 6, we compare the two approaches by means of two numerical examples. Finally, a conclusion is given in Sect. 7.

## DAE setting

This section is devoted to the original formulation of the servo-constraint configuration as a high-index DAE. For this, we consider the dynamics of a mechanical system with servo and possibly holonomic constraints. We start with a prototype of a mechanical system with servo constraints.

### Example 1

**Fig. 1:**
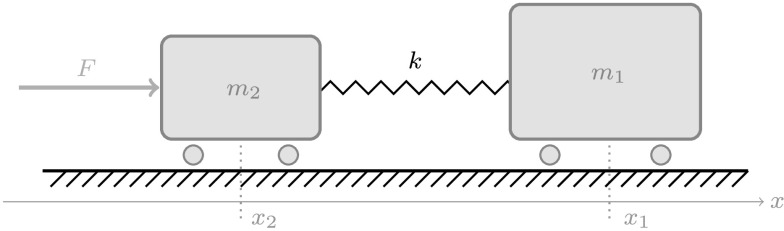
Illustration of the mechanical system from Example 1 including two cars connected by a spring with parameters $k$ and $d$

Consider two cars that are connected via a spring (see Fig. 1), as it was described, e.g., in [8, Sect. II]. This mechanical system is modeled with two degrees of freedom, namely the positions $x_{1}$, $x_{2}$, and one servo constraint. The aim is to make the car on the right follow a desired trajectory given by the function $y$, i.e., to have that $x_{1} = y$. To achieve this, a certain input force $F$ has to be applied. The constrained system has the form

1a $$\begin{aligned} m_{1} \ddot{x}_{1} &= - k (x_{1} - x_{2} - d), \end{aligned}$$

1b $$\begin{aligned} m_{2} \ddot{x}_{2} &= k (x_{1} - x_{2} - d) + F, \end{aligned}$$

1c $$\begin{aligned} x_{1} &= y. \end{aligned}$$

Here, the spring constant $k$ and the spring length $d$ are positive. The given equations of motion, with input $F$ treated as an unknown, form a DAE of (differentiation) index 5. For a definition of the index, we refer to [4, Def. 2.2.2].

Example 1 is a realization of a general second-order system with inputs and servo constraints. In the DAE formulation, where the input is considered as an unknown variable, it reads as follows.

### Configuration 1

For a time interval $[0, T]$, initial values $x^{0}$, $v^{0}\in \mathbb{R}^{n}$ and a forcing term $f\in\mathcal{C}([0,T]; \mathbb{R}^{n})$, for matrices $M\in\mathbb{R}^{n,n}$ symmetric and strictly positive definite and $A \in\mathbb{R}^{n,n}$, for an output operator $C \in\mathbb{R}^{m,n}$ and a desired output $y$ with $y(t)\in\mathbb{R}^{m}$, and for an input operator $B \in\mathbb{R} ^{n,m}$, find an input $u\in\mathcal{C}([0,T];\mathbb{R}^{m})$ and a state trajectory $x \in\mathcal{C}^{2}([0,T];\mathbb{R}^{n})$ such that

2a $$\begin{aligned} M\ddot{x} &= Ax + B u + f, \end{aligned}$$

2b $$\begin{aligned} y &= Cx, \end{aligned}$$

2c $$\begin{aligned} x(0) = x^{0}, \quad &\text{and}\quad\dot{x}(0) = v^{0}. \end{aligned}$$

Another example of Configuration 1 is given by the generalization of Example 1 to a spring-mass chain of $n$ masses connected by $n-1$ springs.

### Example 2

A mass-spring chain, where we want to steer the first mass $x_{1}$ along a trajectory $y$ by applying a force $u$ to the last mass $x_{n}$, can be modeled as in Configuration 1, where $x = [ x_{1}\ x_{2}\ \cdots\ x_{n} ]^{{\mathsf{T}}}$ is the vector of coordinates, $M\in\mathbb{R}^{n,n}$ is the diagonal matrix of the masses, and $A\in\mathbb{R}^{n,n} $, $B\in\mathbb{R}^{n,1} $, $C\in\mathbb{R}^{1,n}$, and $f\in\mathbb{R}^{n,1}$ are given as

3 $$\begin{aligned} &A = \left [ \textstyle\begin{array}{c@{\quad}c@{\quad}c@{\quad}c@{\quad}c@{\quad}c} -k_{1} & k_{1} & \\ k_{1} & -k_{1}-k_{2} & k_{2} \\ & k_{2} & -k_{2}-k _{3} & k_{3} \\ & & \ddots& \ddots& \ddots\\ & & &k_{n-2} &- k_{n-2} -k_{n-1}&k_{n-1} \\ & & & & k_{n-1}&-k_{n-1} \end{array}\displaystyle \right ] , \qquad B = \begin{bmatrix} 0 \\ 0 \\ 0 \\ \vdots\\ 0 \\ 1 \end{bmatrix} , \\ &C = \left[ \textstyle\begin{array}{c@{\quad}c@{\quad}c@{\quad}c} 1 & 0 & \ldots & 0 \end{array}\displaystyle \right] , \quad \text{and}\quad f = \begin{bmatrix} k_{1}d_{1} \\ -k_{1}d_{1} +k_{2}d_{2} \\ \vdots \\ -k_{n-2}d_{n-2} +k_{n-1}d_{n-1} \\ -k_{n-1}d_{n-1} \end{bmatrix} \end{aligned}$$

for given spring constants $k_{1}, \dots, k_{n-1}> 0$ and spring lengths $d_{1}, \dots, d_{n-1} >0$. This system then defines a DAE of index $2n+1$.

In order to model general multibody systems, such as the crane model in [5, Ex. 4], we need to include holonomic constraints of type $g(x)=0$, where $g$ is a suitable possibly nonlinear function. Therefore, we extend Configuration 1 as follows.

### Configuration 2

For a time interval $[0, T]$, initial values $x^{0}$, $v^{0}\in \mathbb{R}^{n}$ and a forcing term $f\in\mathcal{C}([0,T]; \mathbb{R}^{n})$, for $g\colon\mathbb{R}^{n}\to\mathbb{R}^{r}$, for $M\in\mathbb{R}^{n,n}$ symmetric and strictly positive definite and $A \in\mathbb{R}^{n,n}$, for an output operator $C \in\mathbb{R} ^{m,n}$ and a desired output $y$ with $y(t)\in\mathbb{R}^{m}$, and for an input operator $B \in\mathbb{R}^{n,m}$, find an input $u\in \mathcal{C}([0,T];\mathbb{R}^{m})$, a state trajectory $x \in \mathcal{C}^{2}([0,T];\mathbb{R}^{n})$, and a Lagrange multiplier $p \in\mathcal{C}([0,T];\mathbb{R}^{r})$ such that

4a $$\begin{aligned} M\ddot{x} &= Ax + G^{{\mathsf{T}}}(x)p + B u + f, \end{aligned}$$

4b $$\begin{aligned} 0 &= g(x), \end{aligned}$$

4c $$\begin{aligned} y &= Cx, \end{aligned}$$

4d $$\begin{aligned} x(0) = x^{0}, \quad &\text{and}\quad\dot{x}(0) = v^{0}. \end{aligned}$$

Here, the Lagrange multiplier $p$ couples the holonomic constraint (4b) to the dynamical equations (4a), and $G$ is the Jacobian of the holonomic constraint, i.e., $G(x) = \frac{\partial g}{\partial x}(x) \in \mathbb{R}^{r,n}$.

The following observations and assumptions will be formulated for Configuration 2, but they directly apply to Configuration 1 if we omit $g$ and $G^{{\mathsf{T}}}(x)p$. From DAE theory it is known that the derivatives of the right-hand side of system (4a)–(4d) appear in the solution [4, Ch. 2]. Because of the assumed semiexplicit structure of the system, only the derivatives of $g$ and $y$ are part of the solution and, in particular, of the desired input $u$. We can show that in the index-5 case of Example 1, the input depends on the 4th derivative of $y$, whereas for the $n$-spring-mass chain of Example 2, it depends on $y^{(2n)}$. Thus, the following assumptions are made to guarantee a continuous solution $u$ of Configuration 2 or 1.

### Assumption 1

(*DAE setting*) *In the formulation of Configurations* 2 *and* 1, *it is assumed that*:

1. *Smoothness of the data*: $f\in\mathcal{C} ([0,T]; \mathbb {R}^{n})$ *and* $y\in\mathcal{C} ^{\nu_{d}-1}([0,T]; \mathbb{R}^{m})$, *where* $\nu_{d}$ *is the* (*differentiation*) *index of the system equations*.
2. *Consistency of the initial values with respect to the holonomic constraints*: $g(x^{0})=0$ *and* $G(x^{0})v^{0}= 0$, *if applicable*.
3. *Consistency of the initial values with respect to the target output*: $Cx^{0}= y(0)$ *and* $Cv^{0}= \dot{y}(0)$, *and also the conditions that result from the insertion of the differential equation into the servo constraint*,
  $$\begin{aligned} \ddot{y} = C \ddot{x} = CM^{-1} \bigl( Ax + G^{{\mathsf{T}}}(x)p + B u + f\bigr). \end{aligned}$$

### Remark 1

In the $n$-car example from Example 2, the consistency conditions, which directly follow from equation (2b), are $y(0) = x^{0}_{1}$ and $\dot{y}(0) = v ^{0}_{1}$. Furthermore, through the combination of Eqs. (2a) and (2b), we obtain the two conditions

$$\begin{aligned} m_{1} \ddot{y}(0) = k_{1} \bigl(- x^{0}_{1} + x^{0}_{2} + d_{1}\bigr), \qquad m _{1} y^{(3)}(0) = k_{1} \bigl(- v^{0}_{1} + v^{0}_{2}\bigr). \end{aligned}$$

We emphasize that the numerical integration of high-index DAEs involves many difficulties; see, e.g., [21, Ch. II]. Furthermore, the high-index property and the resulting assumptions hypothesize that the DAE formulation (4a)–(4d) does not provide an appropriate model. Thus, we propose a remodeling process that leads to an optimal control problem. This involves a modeling error that is adjustable by a parameter as discussed in the following sections.

### Remark 2

From the observation that the solution of an index-$\nu_{d}$ system depends on the $\nu_{d}$th derivative of the right-hand side it follows that the index is closely related to the so-called *relative degree*; see [18] for a definition. Indeed, for the examples considered in this paper, the rule of thumb that *the relative degree is equal to the index minus 1* applies. However, since the same input-to-output behavior can be realized through DAEs of different indices, this rule does not hold in general.

## Formulation as optimal control problem

Instead of prescribing the servo constraint (4c) in a rigid way, we can formulate it as the target of an optimization problem. Accordingly, the solution does not follow the trajectory exactly, which allows for a less regular target, and which typically leads to smaller input forces.

### Configuration 3

For a $\nu\in\mathbb{N}$, find $u\in\mathcal{C}^{\nu}([0,T]; \mathbb{R}^{m})$ that minimizes the cost functional

5 $$\begin{aligned} \mathcal{J}(x,u) := \mathcal{S}\bigl(x(T)\bigr) + \int_{0}^{T} \mathcal{Q}(x) + \mathcal{R}(u) \, \text{d}t \end{aligned}$$

with the quadratic performance criteria

6a $$\begin{aligned} &\mathcal{Q}(x) : = \frac{1}{2} (Cx-y)^{{\mathsf{T}}}Q (Cx-y), \qquad \mathcal{R}(u):=\frac{1}{2} \sum_{i=0}^{\nu}u^{(i){\mathsf{T}}} R _{i} u^{(i)}, \end{aligned}$$

6b $$\begin{aligned} &\quad\text{and}\quad\mathcal{S}\bigl(x(T)\bigr):= \frac{1}{2} \bigl(Cx(T)-y(T) \bigr)^{{\mathsf{T}}}S \bigl(Cx(T)-y(T) \bigr) \end{aligned}$$

for given $Q\in\mathbb{R}^{m,m} $, $S\in\mathbb{R}^{m,m}$, and $R_{0}, \ldots, R_{\nu}\in\mathbb{R}^{m,m}$ symmetric and positive semidefinite. The state $x=x(u)\in\mathcal{C}^{2}([0,T]; \mathbb{R}^{n})$ is related to $u$ through the dynamics (4a), the holonomic constraint (4b), and the initial conditions (4d).

The parameters $Q$, $S$, and $R_{0}, \ldots, R_{\nu}$ can be chosen to meet certain requirements for the minimization. With this, we may install different kinds of penalizations of the derivatives of this input variable $u$. Note that $R_{0}, \ldots, R_{\nu}$ describe the modeling error compared to the DAE formulation in Configuration 2; see also the discussion in Sect. 4.

Note that in Configuration 3 the fulfillment of the constraint (4c) is balanced with the cost of the input $u$, including its derivatives up to order $\nu$. Also, the necessary smoothness conditions on $y$ for a continuous solution $u$ and the consistency condition of the initial values with respect to the target output (see Assumption 1) can be relaxed.

### Assumption 2

(*Optimal control setting*) *In the formulation of Configuration* 3 *it is assumed that*:

1. *Smoothness of the data*: $f$ *and* $y$ *are continuous on* $[0,T]$.
2. *Consistency of the initial values with respect to the holonomic constraints*: $g(x^{0})=0$ *and* $G(x^{0})v^{0}= 0$, *if applicable*.

### Optimality conditions

We derive *formal* optimality conditions for the optimization problem in Configuration 3; cf. [25, Ch. 6] and [22] for the DAE case with holonomic constraints. In order to apply the standard variational approach, we have to make the following assumption.

#### Assumption 3

*For any input* $u$, *the state equations* (4a)*–*(4b) *with* (4d) *have a unique solution* $x = x(u)$, *and the map* $u \mapsto x(u)$ *that assigns an input* $u$ *to the corresponding solution is differentiable*.

For the considered setups and in particular for the linear problem from Example 2, this assumption is readily confirmed. To derive the formal optimality conditions, we consider the Lagrange functional

7 $$\begin{aligned} \mathcal{L} (u; \lambda, \mu) = \mathcal{J}\bigl(x(u), u\bigr) + \int_{0}^{T} \lambda^{{\mathsf{T}}} \bigl(M\ddot{x} - Ax - G^{{\mathsf{T}}}(x)p - Bu - f \bigr) + \mu^{{\mathsf{T}}}g(x)\,\text{d}t \end{aligned}$$

for formally introduced multipliers $\lambda\in\mathcal{C}^{2}([0, T]; \mathbb{R}^{n})$ and $\mu\in\mathcal{C}([0,T];\mathbb{R}^{r})$.

The formal optimality conditions are derived from the requirement that, for suitable $(\lambda, \mu)$, an optimal $u^{*}$ marks a stationary point of $\mathcal{L}(u;\lambda, \mu)$, i.e.,

8 $$\begin{aligned} \frac{\partial}{\partial u}\mathcal{L}\bigl(u^{*}; \lambda, \mu\bigr)\delta _{u}= 0 \end{aligned}$$

for every variation $\delta_{u}$; cf. [29, Ch. 14]. This is the case if $(x,p,u,\lambda, \mu)$ solves the following formal optimality system.

#### Problem 1

Consider the functions and coefficients defined in Configurations 2 and 3. Find $(x, p)$, $(\lambda, \mu) \in\mathcal{C}^{2}([0,T];\mathbb{R}^{n}) \times\mathcal{C}([0,T];\mathbb{R}^{r})$, and $u \in\mathcal{C}([0,T]; \mathbb{R}^{m})$ such that

9a $$\begin{aligned} M \ddot{x} &= Ax + G^{{\mathsf{T}}}(x)p + B u + f, \end{aligned}$$

9b $$\begin{aligned} 0 &= g(x), \end{aligned}$$

9c $$\begin{aligned} M^{{\mathsf{T}}}\ddot{\lambda} &= A^{{\mathsf{T}}}\lambda+ \frac{ \partial}{\partial x} \bigl(G(x)^{{\mathsf{T}}}p \bigr) \lambda- G^{ {\mathsf{T}}}(x) \mu- C^{{\mathsf{T}}}Q Cx + C^{{\mathsf{T}}}Q y, \end{aligned}$$

9d $$\begin{aligned} 0 &= G(x) \lambda, \end{aligned}$$

9e $$\begin{aligned} 0 &= \sum_{i=0}^{\nu}(-1)^{i} R_{i} u^{(2i)} - B^{{\mathsf{T}}} \lambda \end{aligned}$$

with the initial conditions for $x$ as in Eq. (4d) and the terminal conditions for the dual variable $\lambda$ given by

10 $$\begin{aligned} M^{{\mathsf{T}}}\lambda(T) = 0,\quad M^{{\mathsf{T}}}\dot{ \lambda}(T) = C^{{\mathsf{T}}}S \bigl(C x(T) - y(T)\bigr). \end{aligned}$$

Depending on the parameter $\nu$, we obtain the boundary conditions

11 $$ \sum_{i=1}^{\nu}\sum _{k=1}^{i} (-1)^{k}~ \delta_{u}^{(i-k){\mathsf{T}}}~ R_{i} u^{(i+k-1)} |_{0}^{T} = 0 $$

for all variations $\delta_{u}$ from a suitable subset of $\mathcal{C}([0,T];\mathbb{R}^{m})$.

#### Remark 3

For the case $\nu=0$, by (9e) we obtain the often used algebraic relation

$$\begin{aligned} 0=-B^{{\mathsf{T}}}\lambda+ R_{0}u \end{aligned}$$

and no boundary conditions for the input $u$.

In general, for $\nu>0$, the space of suitable variations $\delta _{u}$ depends on the incorporation of the inputs in the optimality problem. Either, we may add the derivatives of the input to the cost functional as in (5) without any specifications of initial conditions or incorporate the inputs $\dot{u}, \dots, u^{(\nu-1)}$ as part of the state vector as in [25, Rem. 3.8]. In the latter case, initial conditions have to be stated for $u(0), \dots, u^{(\nu-1)}(0)$, which may be unphysical. Furthermore, the corresponding variations $\delta_{u}$, $\dot{\delta}_{u}$, $\ddot{\delta}_{u}, \ldots, \delta_{u}^{( \nu-1)}$ have to vanish at $t=0$.

#### Remark 4

If we do not impose restrictions on admissible inputs $u$ and, thus, on $\delta_{u}$, then for fixed $\nu$ and positive definite $R_{\nu}$, the following conditions need to hold:

$$\begin{aligned} \nu= 1{:} & \qquad \dot{u}(0) = \dot{u}(T) = 0, \\ \nu= 2{:} & \qquad \ddot{u}(0) = \ddot{u}(T) = 0, \\ & \qquad R_{1} \dot{u}(0) - R_{2} u^{(3)}(0) = R_{1} \dot{u}(T) - R_{2} u^{(3)}(T) = 0. \end{aligned}$$

### First-order formulation

In order to apply standard theory and standard numerical routines, we reformulate the optimality system as a first-order system. For this, we restrict ourselves to the unconstrained case $r=0$ with $\nu= 1$. We introduce the variables

$$ z= \begin{bmatrix} x \\ \dot{x} \end{bmatrix} , \qquad \zeta:= \begin{bmatrix} -\dot{\lambda} \\ \lambda \end{bmatrix} , \qquad v := \begin{bmatrix} u \\ -\dot{u} \end{bmatrix} . $$

The DAE (2a)–(2c) in first-order form is given by

12a $$\begin{aligned} \tilde{M}\dot{z} &= \tilde{A}z + \tilde{B}u + \tilde{f}, \end{aligned}$$

12b $$\begin{aligned} \tilde{C}z &= g, \end{aligned}$$

12c $$\begin{aligned} z(0) &= \begin{bmatrix} x^{0}\\ v^{0} \end{bmatrix} \end{aligned}$$

with

$$\begin{aligned} &\tilde{M}:= \left[ \textstyle\begin{array}{c@{\quad}c} I_{n} & 0 \\ 0 & M \end{array}\displaystyle \right] , \qquad\tilde{A}:= \left[ \textstyle\begin{array}{c@{\quad}c} 0 & I_{n} \\ A & 0 \end{array}\displaystyle \right] , \qquad\tilde{B}:= \begin{bmatrix} 0 \\ B \end{bmatrix} , \\ &\tilde{C}:= \left[ \textstyle\begin{array}{c@{\quad}c} C & 0 \end{array}\displaystyle \right] , \qquad\tilde{f}:= \begin{bmatrix} 0 \\ f \end{bmatrix} , \end{aligned}$$

where $I_{n}$ denotes the identity matrix in $\mathbb{R}^{n}$. In order to write the resulting optimality system (9a)–(9e) as a first-order system, we further introduce the matrices

$$\begin{aligned} &\hat{M}^{{\mathsf{T}}}:= \left[ \textstyle\begin{array}{c@{\quad}c} M^{{\mathsf{T}}}& 0\\ 0 & I_{n} \end{array}\displaystyle \right] , \qquad\tilde{J}:= \left[ \textstyle\begin{array}{c@{\quad}c} 0 & I_{m}\\ -I_{m} & 0 \end{array}\displaystyle \right] , \\ &\tilde{I}_{\beta}:= \left[ \textstyle\begin{array}{c@{\quad}c} -\beta_{0}I_{m} & 0\\ 0 & \beta_{1}I_{m} \end{array}\displaystyle \right] , \quad\text{and}\quad \hat{B}:= \left[ \textstyle\begin{array}{c@{\quad}c} \tilde{B} & 0 \end{array}\displaystyle \right] = \left[ \textstyle\begin{array}{c@{\quad}c} 0 & 0 \\ B & 0 \end{array}\displaystyle \right] . \end{aligned}$$

The optimality system with the Lagrange multiplier $\mu$ and control $v$ is given via

13 $$\begin{aligned} \left[ \textstyle\begin{array}{c@{\quad}c@{\quad}c} 0 & \tilde{M}& \\ -\hat{M}^{{\mathsf{T}}}& 0 & \\ & & \beta_{1} \tilde{J} \end{array}\displaystyle \right] \begin{bmatrix} \dot{\zeta}\\ \dot{z} \\ \dot{v} \end{bmatrix} = \left[ \textstyle\begin{array}{c@{\quad}c@{\quad}c} 0 & \tilde{A}& \hat{B}\\ \tilde{A}^{{\mathsf{T}}}& -\tilde{C}^{{\mathsf{T}}} \tilde{C}& \\ \hat{B}^{{\mathsf{T}}}& & \tilde{I}_{\beta} \end{array}\displaystyle \right] \begin{bmatrix} \zeta\\ z \\ v \end{bmatrix} + \begin{bmatrix} \tilde{f}\\ \tilde{C}^{{\mathsf{T}}}g \\ 0 \end{bmatrix} \end{aligned}$$

with the initial and terminal conditions

14 $$\begin{aligned} z(0) = \begin{bmatrix} x^{0}\\ v^{0} \end{bmatrix} , \qquad -\hat{M}^{{\mathsf{T}}}\zeta(T) = \gamma\tilde{C}^{{\mathsf{T}}}\bigl( \tilde{C}x(T) - g(T)\bigr), \qquad\dot{u}(0) = 0,\qquad\dot{u}(T) = 0. \end{aligned}$$

In the case where an initial condition for the input was prescribed, i.e., $u(0) = u^{0}$ is given, the conditions for $u$ in (14) reduce to $\dot{u}(T) = 0$. With appropriate matrices $\mathcal{B} _{0}$ and $\mathcal{B} _{T}$ and a vector $\rho\in\mathbb{R}^{4n+2m}$, these boundary conditions can also be written in the form

$$\begin{aligned} \mathcal{B} _{0} \begin{bmatrix} \zeta(0) \\ z(0) \\ v(0) \end{bmatrix} + \mathcal{B} _{T} \begin{bmatrix} \zeta(T) \\ z(T) \\ v(T) \end{bmatrix} = \rho. \end{aligned}$$

This formulation is used in Sect. 5.2.2.

### Necessary conditions for the existence of an optimal solution

In this subsection, the particular aspect of *consistency* as it arises in the context of optimal control of DAEs is discussed. Also, a mathematical result that can be used two overcome this *consistency* issue in the case of optimal control of multibody systems with possibly nonlinear holonomic constraints is presented.

If the optimality system (9a)–(11) has a solution, then it provides necessary optimality conditions for $(x(u),u)$. However, in the considered DAE context, i.e., when holonomic constraints are applied, it may happen that the optimization problem has a solution whereas the *formal* optimality system is not solvable [23]. Apart from the general case that the boundary values do not permit a solution [2], for a DAE, a solution may not exist because of insufficient smoothness of the data or because of inconsistent initial or terminal values.

Thus, it is an important task to establish necessary conditions for solvability of the formal optimality system. By Assumption 2 the initial conditions for $x$ are consistent. The adjoint equations (9b) and (9d) have the same differential-algebraic structure, so that from (9d) we can read off the consistency conditions for the terminal values for $\lambda$, namely

15 $$ G\bigl(x(T)\bigr)\lambda(T) = 0 \quad\text{and} \quad \frac{\text{d}}{ \text{d}t} \bigl(G\bigl(x(T)\bigr) \bigr)\lambda(T) + G\bigl(x(T)\bigr) \dot{\lambda}(T) = 0; $$

cf. Assumption 2(2). Comparing the prescribed terminal conditions (10) for $\lambda$ to (15), we obtain the necessary and sufficient condition for consistency as

16 $$ 0 = G\bigl(x(T)\bigr) M^{-{\mathsf{T}}}C^{{\mathsf{T}}}S \bigl(C x(T) - y(T)\bigr). $$

Similar conditions in a slightly different formulation have been reported in [26], where the authors proposed the variants to remove the end point penalization from the cost functional or to consider a regularization of the dynamical equation. Within this regularization, the constraint (4b) is replaced by its derivative.

The following theorem shows that, instead of the state equations, we can modify the cost functional. This modification ensures consistency while affecting neither the performance criterion nor the necessity of the formal optimal conditions.

#### Theorem 1

(*Ensuring consistency*) *Let* $P_{x^{*}(T)}$ *be a projector onto the kernel of* $G(x^{*}(T))$ *that satisfies* $M^{-{\mathsf{T}}}P_{x^{*}(T)}^{{\mathsf{T}}}=P _{x^{*}(T)}M^{-{\mathsf{T}}}$. *Then*, *replacing the terminal conditions* (10) *for* $\lambda$ *by the conditions*

17 $$\begin{aligned} M^{{\mathsf{T}}}\lambda(T) = 0,\qquad M^{{\mathsf{T}}}\dot{\lambda}(T) = P_{x^{*}(T)}C^{{\mathsf{T}}}S \bigl(C x^{*}(T) - y(T)\bigr) \end{aligned}$$

*ensures consistency of the terminal conditions for* $\lambda$. *Moreover*, *if* $(x^{*},p^{*},u^{*},\lambda,\mu)$ *solve the optimality system with* (17), *then* $u^{*}$ *is a stationary point of* (7).

#### Proof

Let $u^{*}$ be a solution to the optimality system and consider the first variation $\frac{\partial}{\partial u}\mathcal{L}(u^{*};\lambda , \mu)$. The relation that defines the terminal condition for $\dot{\lambda}^{{\mathsf{T}}}$ is given by

18 $$\begin{aligned} 0 =& \frac{\partial}{\partial u} \bigl(\mathcal{S}\bigl(x^{*}(T) \bigr) \bigr) \delta_{u}(T) - \dot{\lambda}^{{\mathsf{T}}}(T) \delta_{x(\delta_{u})}(T) \\ = & \frac{\partial}{\partial x} \bigl(S\bigl(x^{*}(T)\bigr) \bigr) \delta_{x(\delta_{u})}(T)-\dot{\lambda}^{{\mathsf{T}}}(T) \delta_{x(\delta_{u})}(T), \end{aligned}$$

where $\delta_{x(\delta_{u})}= \frac{\partial}{\partial u} (x ^{*}(u) )\delta_{u}$ is the variation in $x^{*}$ induced by the variation of $u$. Since $\delta_{x(\delta_{u})}$ solves the state equations (4a)–(4b) linearized about $x^{*}$ with input $\delta_{u}$ (cf. [28, Ch. 2]), we have that $\delta_{x(\delta_{u})}(T)$ fulfills the linearized constraint (4b), i.e., $G(x^{*}(T))\delta_{x(\delta_{u})}(T) = 0$ or, equivalently, $\delta_{x(\delta_{u})}(T)=P_{x^{*}(T)} \delta_{x(\delta_{u})}(T)$. Accordingly, relation (18) does not change if we replace $\frac{\partial}{\partial x} (S(x^{*}(T)) )$ by $\frac{ \partial}{\partial x} (S(x^{*}(T)) )P_{x^{*}(T)}$. For the considered quadratic cost functional (5), this means that the formal conditions (16) are equivalent (in the sense that the first variation of ℒ is not affected) to

$$\begin{aligned} \dot{\lambda}(T) = P_{x^{*}(T)}\frac{\partial}{\partial x} \bigl( \mathcal{S} \bigl(x^{*}(T)\bigr) \bigr)^{{\mathsf{T}}}= P_{x^{*}(T)}C^{{\mathsf{T}}}S \bigl(C x^{*}(T) - y(T)\bigr), \end{aligned}$$

which concludes the proof. □

#### Remark 5

In the general case, $P_{x^{*}(T)}$ is defined implicitly since it depends on the unknown solution $x^{*}$. In the case of linear holonomic constraints, $P_{x^{*}(T)}$ is readily computed; see [15, Rem. 8.20]. As in the example presented further, in order to ensure consistency of the terminal conditions, we may also use a projection onto a subspace of $G(x(T))$ that is possibly independent of $x$. This, however, will effectively alter the performance criterion $\mathcal{S}$.

#### Remark 6

If $M$ is symmetric, then the condition $M^{-{\mathsf{T}}}P_{x^{*}(T)} ^{{\mathsf{T}}}=P_{x^{*}(T)}M^{-{\mathsf{T}}}$ is the orthogonality condition in the inner product induced by $M^{-1}$, which is the natural inner product in PDE applications.

### Existence of optimal solutions

For Configuration 3 constrained by linear equations without holonomic constraints as in (2a), the existence of solutions is provided by well-known results.

#### Lemma 1

(*Existence of an optimal solution*) *For* $\nu\geq0$, *consider the optimal control problem with cost functional* (5) *constrained by* (2a) *and let Assumption* 2 *hold*. *If* $R_{\nu}> 0$ *and if* $u(0)$, $\dot{u}(0), \ldots, u^{(\nu-1)}(0)$ *are given*, *then system* (9a)*–*(9e) *and the optimal control problem have a unique solution for any* $T<\infty$ *and initial data* $x^{0}$ *and* $v^{0}$.

#### Proof

Recall that, by the standard order reduction approach, the second-order system (9a)–(9e) can be reformulated as an equivalent first-order system; see Sect. 3.2.

Then, for $\nu=0$, the result is given in [25, Rem. 3.6]. For $\nu=1$ with $R_{1} >0$, we may introduce a new variable for the derivative of the control $u$. Interpreting $u$ as a part of the state variable whereas its derivative $v:= \dot{u}$ is the new control variable, the same arguments apply; cf. [25, Rem. 3.8]. Note that this ansatz requires an initial value for $u$. This procedure may be successively repeated for $\nu> 1$. □

#### Remark 7

Note that the existence result in Lemma 1 is true for all initial values $x^{0}$ and $v^{0}$ in contrast to the DAE (4a)–(4d), which requires consistent initial data. The case $\nu=0$ with $R_{0}= 0$ yields again the DAE formulation of the problem and thus, needs consistent boundary conditions. This case is discussed in Sect. 4.

For the nonlinear optimality system (9a)–(9e) with holonomic constraints, we use the strong but reasonable assumption that the state equations (4a)–(4d) have a solution for any input $u$ under the consideration that the solutions of the state equations depend smoothly on the input (Assumption 3) to state that existence of a solution to the formal optimality system is indeed a necessary condition for optimality.

We first show that the adjoint equations have a solution for every state trajectory and, thus, also at the optimal solution. Then we confer that the smoothness of the input to state map implies that, at an optimal solution, the gradient condition (9e) must also be fulfilled.

#### Lemma 2

*Consider a solution* $(x, p)$ *of* (4a)*–*(4d). *If* $g$ *is sufficiently smooth and* $G(x(t))$ *has full row rank for all* $t\in[0,T]$ *and if the end condition*

19 $$\begin{aligned} M^{{\mathsf{T}}}\lambda(T) = 0,\qquad M^{{\mathsf{T}}}\dot{\lambda}(T) = C^{{\mathsf{T}}}S \bigl(C x(T) - y(T)\bigr) \end{aligned}$$

*is consistent*, *then the adjoint equations* (9c)*–*(9d) *with end condition* (19) *have a unique solution*.

#### Proof

We rewrite the adjoint equations as the first-order system (cf. the second equation of (13))

20a $$\begin{aligned} \left[ \textstyle\begin{array}{c@{\quad}c} M^{{\mathsf{T}}}& 0 \\ 0 & M^{{\mathsf{T}}} \end{array}\displaystyle \right] \frac{\text{d}}{\text{d}t} \begin{bmatrix} \lambda\\ \dot{\lambda} \end{bmatrix} &= \left[ \textstyle\begin{array}{c@{\quad}c} 0 & M^{{\mathsf{T}}} \\ \tilde{A}^{{\mathsf{T}}} & 0 \end{array}\displaystyle \right] \begin{bmatrix} \lambda\\ \dot{\lambda} \end{bmatrix} + \begin{bmatrix} 0 \\ G^{{\mathsf{T}}} \end{bmatrix} \mu+ \begin{bmatrix} 0 \\ \tilde{f} \end{bmatrix} , \end{aligned}$$

20b $$\begin{aligned} G\lambda &= 0, \end{aligned}$$

where $\mu$ is the multiplier that accounts for the holonomic constraint in (7). Therein, the dependencies on $x$ and $t$ were omitted, and all linear coefficients and inhomogeneities in $\tilde{A}$ and $\tilde{f}$ were clustered. Then, via another multiplier $\eta$, the differentiated constraint $\dot{G} \lambda+ G \dot{\lambda}= 0$ is added to the system and yields

21a $$\begin{aligned} \left[ \textstyle\begin{array}{c@{\quad}c} M^{{\mathsf{T}}}& 0 \\ 0 & M^{{\mathsf{T}}} \end{array}\displaystyle \right] \frac{\text{d}}{\text{d}t} \begin{bmatrix} \lambda\\ \dot{\lambda} \end{bmatrix} &= \left[ \textstyle\begin{array}{c@{\quad}c} 0 & M^{{\mathsf{T}}}\\ \tilde{A}^{{\mathsf{T}}}& 0 \end{array}\displaystyle \right] \begin{bmatrix} \lambda\\ \dot{\lambda} \end{bmatrix} + \left[ \textstyle\begin{array}{c@{\quad}c} G^{{\mathsf{T}}}&\dot{G}^{{\mathsf{T}}}\\ 0 & G^{{\mathsf{T}}} \end{array}\displaystyle \right] \begin{bmatrix} \eta\\ \mu \end{bmatrix} + \begin{bmatrix} 0 \\ \tilde{f} \end{bmatrix} , \end{aligned}$$

21b $$\begin{aligned} \left[ \textstyle\begin{array}{c@{\quad}c} G & 0 \\ \dot{G} & G \end{array}\displaystyle \right] \begin{bmatrix} \lambda\\ \dot{\lambda} \end{bmatrix} &= 0. \end{aligned}$$

Since $G$ has pointwise full row rank and since the terminal conditions are assumed to be consistent, by [15, Thm. 8.6] we can state that system (21a)–(21b) has a unique solution. We can show that the parts $(\lambda, \mu)$ of a solution $(\lambda, \eta, \mu)$ to (21a)–(21b) also solve (20a)–(20b). The other way round, by construction and by the smoothness assumption on $G$, a solution $(\lambda, \mu)$ to (20a)–(20b) partially defines a solution to (21a)–(21b) and, thus, is unique. □

#### Theorem 2

*Assume that* $u\mapsto x$ *is Lipschitz continuous*. *If for* $(x(u_{0}), u _{0})$, *the constraints and the cost functional are Gâteaux differentiable with respect to* $x$ *at* $x(u_{0})$ *and if the terminal conditions* (10) *are consistent*, *then the optimality system* (9a)*–*(9e) *is a necessary condition for optimality of* $(x(u_{0}), u_{0})$.

#### Proof

At every candidate solution $x(u_{0})$, the adjoint equations (9c) and (9d) with (10) are solvable by Lemma 2. Then, the claim follows from the result given in [15, Thm. 5.5]. □

Concerning sufficiency for the existence of unique global or local solutions, general results for constrained optimization extended to optimal control problems can be consulted; see, e.g., [15, Ch. 5.3].

### Various optimality systems

In the remaining part of this section, we consider particular cases of the optimality system (9a)–(9e) for constrained and nonconstrained systems and different values of $\nu$. For this, consider again the cost functional (5) and set

$$\begin{aligned} Q = I_{m}, \qquad R_{i} = \beta_{i} I_{m}, \qquad S = \gamma I_{m}. \end{aligned}$$

Thus, the remaining parameters for the optimization problem are $\gamma$, $\beta_{0}, \dots, \beta_{\nu}\ge0$. For different values of $\nu$, the various structures of the optimality system are analyzed. Note that this subsection is restricted to $\beta_{\nu}> 0$. The particular case $\nu=0$ with $\beta_{0}=0$, i.e., with no constraints on the input at all, is then discussed in Sect. 4.

#### Case $r=0$, $\nu=0$

Consider the case of Configuration 3 with $r=0$, i.e., the optimization is constrained by an ODE instead of a DAE. As mentioned before, it is assumed here that $\beta_{0} > 0$. This then leads to the optimality system

$$\begin{aligned} \left[ \textstyle\begin{array}{c@{\quad}c@{\quad}c} 0 & M & \\ M^{{\mathsf{T}}}& 0 & \\ & & 0 \end{array}\displaystyle \right] \begin{bmatrix} \ddot{\lambda}\\ \ddot{x} \\ \ddot{u} \end{bmatrix} = \left[ \textstyle\begin{array}{c@{\quad}c@{\quad}c} 0 & A & B \\ A^{{\mathsf{T}}}& -C^{{\mathsf{T}}}C & \\ -B^{{\mathsf{T}}}& & \beta_{0} I_{m} \end{array}\displaystyle \right] \begin{bmatrix} \lambda\\ x \\ u \end{bmatrix} + \begin{bmatrix} f \\ C^{{\mathsf{T}}}y\\ 0 \end{bmatrix} . \end{aligned}$$

Thus, we obtain a DAE of index 1 but with initial and terminal conditions of the form

$$\begin{aligned} x(0) = x^{0},\qquad\dot{x}(0) = v^{0}, \qquad\lambda(T) = 0, \qquad M ^{{\mathsf{T}}}\dot{\lambda}(T) = \gamma C^{{\mathsf{T}}}\bigl(Cx(T) - y(T) \bigr). \end{aligned}$$

#### Case $r=0$, $\nu=1$

For the same case but with the inclusion of a penalization of the first derivative of the input $u$ with parameter $\beta_{1} \neq 0$, the optimality system (9a)–(9e) reads

22 $$\begin{aligned} \left[ \textstyle\begin{array}{c@{\quad}c@{\quad}c} 0 & M & \\ M^{{\mathsf{T}}}& 0 & \\ & & \beta_{1} I_{m} \end{array}\displaystyle \right] \begin{bmatrix} \ddot{\lambda}\\ \ddot{x} \\ \ddot{u} \end{bmatrix} = \left[ \textstyle\begin{array}{c@{\quad}c@{\quad}c} 0 & A & B \\ A^{{\mathsf{T}}}& -C^{{\mathsf{T}}}C & \\ -B^{{\mathsf{T}}}& & \beta_{0} I_{m} \end{array}\displaystyle \right] \begin{bmatrix} \lambda\\ x \\ u \end{bmatrix} + \begin{bmatrix} f \\ C^{{\mathsf{T}}}y\\ 0 \end{bmatrix} . \end{aligned}$$

In contrast to the case with $\nu= 0$, the leading matrix on the left-hand side is invertible. Thus, the optimality system (22) is an ODE. The corresponding boundary conditions are given by

$$\begin{aligned} &x(0) = x^{0},\qquad\dot{x}(0) = v^{0}, \qquad\dot{u}(0) = 0, \qquad \dot{u}(T) = 0, \\ &\lambda(T) = 0,\qquad M^{{\mathsf{T}}}\dot{\lambda}(T) = \gamma C ^{{\mathsf{T}}}\bigl(Cx(T) - y(T)\bigr). \end{aligned}$$

#### Case $r=0$, $\nu=2$

In the case $\nu=2$, the second derivatives of the inputs are also penalized, i.e., $\beta_{2} \neq 0$. As seen in Eq. (9e), the fourth derivative of $u$ appears in the optimality system. In order to write the system in a second-order form, we introduce a new variable $v:=\ddot{u}$. The optimality system then has the form

23 $$\begin{aligned} \left[ \textstyle\begin{array}{c@{\quad}c@{\quad}c@{\quad}c} 0 & M & & \\ M^{{\mathsf{T}}}& 0 & & \\ & & 0 & \beta_{2} I_{m} \\ & & I_{m} & 0 \end{array}\displaystyle \right] \begin{bmatrix} \ddot{\lambda}\\ \ddot{x} \\ \ddot{u} \\ \ddot{v} \end{bmatrix} = \left[ \textstyle\begin{array}{c@{\quad}c@{\quad}c@{\quad}c} 0 & A & B & \\ A^{{\mathsf{T}}}& -C^{{\mathsf{T}}}C & & \\ B^{{\mathsf{T}}}& & -\beta_{0} I_{m} & \beta_{1} I_{m} \\ & & 0 & I _{m} \end{array}\displaystyle \right] \begin{bmatrix} \lambda\\ x \\ u \\ v \end{bmatrix} + \begin{bmatrix} f \\ C^{{\mathsf{T}}}y\\ 0 \\ 0 \end{bmatrix} . \end{aligned}$$

This is again an ODE, and the corresponding boundary values read:

$$\begin{aligned} &x(0) = x^{0},\qquad\dot{x}(0) = v^{0}, \qquad\ddot{u}(0) = 0,\qquad \ddot{u}(T) = 0, \\ &\beta_{1}\dot{u}(0) = \beta_{2}\dot{v}(0),\qquad \beta_{1}\dot{u}(T) = \beta_{2} \dot{v}(T), \\ &\lambda(T) = 0, \qquad M^{{\mathsf{T}}}\dot{\lambda}(T) = \gamma C ^{{\mathsf{T}}}\bigl(Cx(T) - y(T)\bigr). \end{aligned}$$

#### Case $r>0$, $\nu=0$

Finally, we give an example of the optimality system if the cost functional is constrained by a DAE. Here, no derivatives of the inputs are penalized, i.e., $\nu=0$ and $\beta_{0}\neq 0$. Assuming that the Jacobian $G$ is constant, i.e., Eq. (9b) is of the form $0=g(x) = G x$, we obtain the optimality system

$$\begin{aligned} &\left[ \textstyle\begin{array}{c@{\quad}c@{\quad}c@{\quad}c@{\quad}c} 0 & M & & & \\ M^{{\mathsf{T}}}& 0 & & & \\ & & 0 & & \\ & & & 0 & \\ & & & & 0 \end{array}\displaystyle \right] \begin{bmatrix} \ddot{\lambda}\\ \ddot{x} \\ \ddot{p} \\ -\ddot{\mu}\\ -\ddot{u} \end{bmatrix} \\ &\quad = \left[ \textstyle\begin{array}{c@{\quad}c@{\quad}c@{\quad}c@{\quad}c} 0 & A & G^{{\mathsf{T}}}& 0 & -B \\ A^{{\mathsf{T}}}& -C^{{\mathsf{T}}}C & 0 & G^{{\mathsf{T}}}& 0 \\ G & 0 & 0 & & \\ 0 & G & & 0 & \\ -B^{ {\mathsf{T}}}& 0 & & & -\beta_{0} I_{m} \end{array}\displaystyle \right] \begin{bmatrix} \lambda\\ x \\ p \\ -\mu\\ -u \end{bmatrix} + \begin{bmatrix} f \\ C^{{\mathsf{T}}}y\\ 0 \\ 0 \\ 0 \end{bmatrix} . \end{aligned}$$

In contrast to the previous cases, this is a DAE of index 3, but again with a mixture of initial and terminal conditions. This can be seen by the particular structure of the system, which is the same as that for constrained multibody systems; cf. [17, Ch. VII.1]. This is no surprise since only the “index-5 constraint” was removed by the cost functional.

## Comparison of DAE and optimal control solutions

To discuss the qualitative behavior of the solutions of the optimal control problem, we consider the linear case without holonomic constraints (Configuration 1) and, in particular, discuss the $n$-element mass-spring chains as in Example 2.

In the optimal control setting of Sect. 3, it is reasonable to assume that $R_{\nu}$ is positive definite. In the sequel, we analyze the limit case with $\nu=0$ and $R_{0}=0$, i.e., the case in which the control is not constrained at all.

### Equivalence for $R_{0}=0$

We show that for Example 2 with $\nu=0$ and $R_{0}=0$, the DAE approach of Configuration 1 is equivalent to the optimal control formulation in Configuration 3, provided that $Q>0$. Note that this implies that the corresponding optimality system is only solvable for $y\in\mathcal{C} ^{2n}([0,T]; \mathbb{R})$. Recall that $n$ denotes the number of coupled cars.

It is easy to see that a solution $(x,u)$ of the original DAE (2a)–(2c) minimizes the cost functional for $R_{0}=0$. For a solution $x$ of the DAE, we have $Cx=y$ such that the cost functional $\mathcal{J} $ from (5) is minimized since

$$\begin{aligned} \mathcal{J} (x,u) = \mathcal{S}\bigl(x(T)\bigr) + \frac{1}{2} \int_{0}^{T} (Cx-y)^{ {\mathsf{T}}}Q (Cx-y) \, \text{d}t= 0. \end{aligned}$$

Let us consider the optimality system for the case $R_{0}=0$. Equation (9e) reduces to $0 = B^{{\mathsf{T}}}\lambda$, which directly implies that the last component of $\lambda$ vanishes, i.e., $\lambda_{n}=0$. As a result, Eq. (9c) has the form

$$\begin{aligned} \begin{bmatrix} \ddot{\lambda}_{1} \\ \ddot{\lambda}_{2} \\ \vdots \\ \ddot{\lambda}_{n-1} \\ 0 \end{bmatrix} = \left[ \textstyle\begin{array}{c@{\quad}c@{\quad}c@{\quad}c@{\quad}c} -k_{1} & k_{1} & \\ k_{1} & -k_{1}-k_{2} & k_{2} \\ & \ddots& \ddots& \ddots \\ & &k_{n-2} &- k_{n-2} -k_{n-1}&k_{n-1} \\ & & & k_{n-1}&-k_{n-1} \end{array}\displaystyle \right] \begin{bmatrix} \lambda_{1} \\ \lambda_{2} \\ \vdots \\ \lambda_{n-1} \\ 0 \end{bmatrix} - \begin{bmatrix} Q(x_{1}-y) \\ 0 \\ \vdots \\ 0 \\ 0 \end{bmatrix} . \end{aligned}$$

In agreement with the boundary conditions of $\lambda$, we obtain successively $\lambda_{n-1} = \cdots= \lambda_{1} = 0$. If $Q$ is invertible, then this implies that $x_{1}=Cx=y$, which then resembles Configuration 1. In this case, also condition (10) is satisfied, and, thus, Configurations 1 and 3 are equivalent. If $Q$ is not invertible, then the system is not uniquely solvable.

#### Remark 8

The preceding observation is an instance of the general fact that if the linear system without holonomic constraints is controllable and observable and if $Q$ is invertible, then, provided that the data is sufficiently smooth, a solution to the optimal control problem (Configuration 3) resembles the solution of the DAE of Configuration 1. To see this, recall that in the considered situation, the system is observable if and only if $Cx-y=0$ implies $x_{1}=y$, and, by duality, that the system is controllable if and only if $B^{{\mathsf{T}}}\lambda= 0$ implies that $\lambda= 0$ for all time.

#### Remark 9

The equivalence of the DAE and optimal control approach for $R_{0}=0$ can also be shown for the overhead crane from the example in Sect. 6.3, which includes a holonomic constraint. In this case, we can show in a similar manner that the dual variables $\lambda$ and $\mu$ vanish, so that the servo constraint $Cx=y$ has to be satisfied.

### Convergence barriers

By Lemma 1, if $y\in\mathcal{C}([0,T], \mathbb{R})$ and $R_{0}>0$, then Configuration 3 with $\nu=0$, subject to linear constraints, has a unique solution. By the results of the previous Sect. 4.1, for $R_{0}=0$, a solution only exists if $y$ sufficiently smooth.

In this subsection, we examine how the optimal control $u$ behaves when $R_{0} \to0$ in dependence of the smoothness of $y$. Consider the $n$-car example from Example 2 and the associated adjoint equations

24 $$ M^{{\mathsf{T}}}\ddot{\lambda}= A^{{\mathsf{T}}}\lambda- C^{{\mathsf{T}}}(Cx-y) $$

with $A$ and $C$ from (3). With

25 $$ \Delta_{j} := k_{j} \biggl(\frac{\lambda_{j}}{m_{j}}- \frac{\lambda_{j-1}}{m_{j-1}} \biggr), \quad j=2,3,\ldots,n, $$

we can rewrite Eq. (24) as

26a $$\begin{aligned} \ddot{\lambda}_{1} &= - \Delta_{2} - (x_{1}-y), \end{aligned}$$

26b $$\begin{aligned} \ddot{\lambda}_{2} &= \Delta_{2} - \Delta_{3}, \end{aligned}$$

26c $$\begin{aligned} &\ \vdots \\ \ddot{\lambda}_{n-1} &= \Delta_{n} - \Delta_{n-1}, \end{aligned}$$

26d $$\begin{aligned} \ddot{\lambda}_{n} &= \Delta_{n} . \end{aligned}$$

The gradient condition (9e) gives $\lambda_{n}=R _{0}u$ and $\Delta_{n}=\ddot{\lambda}_{n} = R_{0}\ddot{u}$. By a combination of (25) and (26a)–(26d) we recursively compute $\lambda_{n-1}, \lambda_{n-2}, \ldots, \lambda_{1}$ via the formulas

$$\begin{aligned} \lambda_{j-1} =-\frac{1}{k_{j-1}}\Delta_{j}+ \frac{m_{j-1}}{m_{j}} \lambda_{j}\quad\text{and} \quad \Delta_{j-1}=\Delta_{j}- \ddot{\lambda}_{j-1}. \end{aligned}$$

Finally, via (26a), we can directly relate the difference in the target $x_{1}-y$ to the computed input $u$. Assuming uniform masses $m$ and uniform spring constants $k$, for $n=2$, we find

27 $$ x_{1}-y = R_{0} \biggl(2\ddot{u} - \frac{1}{k}u^{(4)} \biggr), $$

whereas for $n=3$ it must hold that

28 $$ x_{1}-y = R_{0} \biggl( \biggl( \frac{1}{k}+1\biggr)u^{(4)}-\frac{1}{k} \biggl( \frac{1}{k} + 1\biggr)u^{(6)} \biggr). $$

We observe that

1. For nonsmooth $y$, where we cannot expect the convergence of $x_{1}$ to $y$, the control $u$ has strong peaks in its derivatives in order to fulfill (27) or (28) as $R_{0} \to0$.
2. For moderate values of $R_{0}$, the tracking error $x_{1}-y$ is affected by the oscillations in the derivatives of $u$ multiplied by multiples of $\frac{1}{k}$ depending on the length of the considered chain.
3. As $k\to\infty$, i.e., when the connections between the cars become more rigid, the higher derivatives of $u$ are damped out from the tracking error. In fact, if one connection is rigid, then the two connected cars can be considered as one, and the index of the system reduces.

## Solution strategies

Within this section, we review several concepts how to solve numerically mechanical systems with servo constraints. First, we comment on the classical approach where the model is given by the DAE (4a)–(4d). In this case, index reduction methods are applied, which then allow us to integrate the resulting equations. Second, using the optimal control ansatz (5), we consider the two cases of either solving directly the optimality system, which is a boundary value problem (BVP), or the resulting Riccati equations. The latter approach may then be used to define a feedback control.

### Solving high-index DAEs

As mentioned already in the introduction, the computation of the inverse dynamics of a discrete mechanical system given by a specification of a trajectory is a highly challenging problem [7, 8]. The reason is the high-index structure of the resulting DAEs. In the case of underactuated mechanical systems considered here, the systems are often of (differentiation) index 5 but may be arbitrarily high as shown in Example 2.

In order to realize the so-called *feedforward control* of the system, we have to solve this high-index DAE. Here, it is advisable to apply index reduction methods instead of solving the equations directly [4, Ch. 5.4]. A well-known approach based on a projection of the dynamics was introduced in [8]; see also [6]. For this, we have to compute time-dependent projection matrices in order to split the dynamics of the underactuated system into constrained and unconstrained parts.

Instead, we may also use the index reduction technique called *minimal extension* [20]. This technique profits from the given semiexplicit structure of the dynamical system and can be easily applied. The application to a wide range of crane models can be found in the recent papers [1, 3]. Therein, it is shown that the method of minimal extension may even be applied the second time, which then leads to a DAE of index 1 for which the numerical integration works essentially as for stiff ODEs [17, Ch. VI.1].

We remark that index reduction techniques are inevitable for numerical simulations of high-index problems. However, for applications like the $n$-car example given in the introduction, which is of index $2n+1$, the DAE approach does not seem to be applicable. The modeling presented here as an optimal control problem still works properly for the general case. For a numerical example including a 3-car model, we refer to Sect. 6.

### Direct solution of the optimality BVP

In this subsection, we discuss the application of the finite difference method and shooting approach in order to solve the optimality system (9a)–(9e).

#### Finite differences

The optimality system includes both initial and terminal conditions, so that the application of standard time-stepping methods is not possible. A straight-forward approach is to introduce a grid of the time domain and to apply the method of finite differences to the differential coupled equations, which leads to a (large but block-sparse) algebraic system. Alternatively, we can apply finite elements or more general collocation methods.

#### Shooting method

For the application of the shooting method, we consider the first-order system (13), i.e., we consider again the case with $r=0$ (no additional holonomic constraint) and $\nu=1$.

For notational reasons, we write system (13) shortly as $\mathcal{M} \dot{y} = \mathcal{K} y + h$, i.e., the vector $y$ includes all state, input, and dual variables. Recall that the boundary conditions can be written in the form $\mathcal{B} _{0} y(0) + \mathcal{B} _{T} y(T) = \rho$. Because of the special structure of the given boundary conditions (initial and terminal conditions are not mixed), we may assume a reordering of the variables in order to get a system of the form

$$\begin{aligned} \mathcal{M} \dot{y} = \mathcal{K} y + h, \qquad \begin{bmatrix} \mathcal{B} _{01} \\ 0 \end{bmatrix} y(0) + \begin{bmatrix} 0 \\ \mathcal{B} _{T2} \end{bmatrix} y(T) = \begin{bmatrix} \rho_{1} \\ \rho_{2} \end{bmatrix} . \end{aligned}$$

The aim of the shooting method is to restore the initial conditions for the entire vector $y$ such that methods for initial value problems are applicable again. Since the initial values of the state variables and $\dot{u}$ (respectively, $u$ if initial data for the input were prescribed) are already given, we can apply the so-called *reduced superposition* [2, Ch. 4.2.4]. This reduces the computational effort of the method. Within the following algorithm, we denote by $s$ the size of the original system and, thus, by $2s$ the size of the first-order system we want to solve.

*Step 1:* Search for the fundamental solution of the corresponding homogeneous system. However, using the reduced superposition, it is sufficient to compute $Y \in\mathbb{R}^{2s,s}$ solving

$$\begin{aligned} \mathcal{M} \dot{Y} = \mathcal{K} Y, \qquad Y(0) = \left[ \textstyle\begin{array}{c@{\quad}c@{\quad}c@{\quad}c@{\quad}c@{\quad}c} I & & 0 & & & \\ & I & & 0 & & \\ & & & & I & 0 \end{array}\displaystyle \right] ^{{\mathsf{T}}}. \end{aligned}$$

*Step 2:* Find a solution $w\in\mathbb{R}^{2s}$ of the initial value problem

$$\begin{aligned} \mathcal{M} \dot{w} = \mathcal{K} w + h, \qquad w(0) = \left[ \textstyle\begin{array}{c@{\quad}c@{\quad}c@{\quad}c@{\quad}c@{\quad}c} 0 & 0 & x^{0}& v^{0}& 0 & 0 \end{array}\displaystyle \right] ^{{\mathsf{T}}}. \end{aligned}$$

*Step 3:* Find the coefficients $c\in\mathbb{R}^{s}$ given by the linear system

$$\begin{aligned} \mathcal{B} _{T2} Y(T) c = \rho_{2} - \mathcal{B} _{T2} w(T), \end{aligned}$$

which then gives the solution of the BVP as $y = Yc + w$. Thus, an approximation of $y$ can be either given directly if the matrices $Y$ and $w$ were stored on the entire time grid or by solving the IVP

$$\begin{aligned} \mathcal{M} \dot{y} = \mathcal{K} y + h \quad\mbox{with } y(0) = Y(0)c + w(0). \end{aligned}$$

##### Remark 10

(Comparison of computational effort) Assume that we always use the same time step size with $N$ grid points. Then, the finite difference method leads to a system of size $2sN$ such that the computational effort is quadratic in $N$. For the shooting method, we have to solve several initial value problems (each using $N$ time steps). Note that the size of the systems is bounded by the size of the original BVP such that the overall costs are only linear in $N$ (but with a large constant depending on $s^{2}$).

##### Remark 11

A more stable extension of the (single) shooting method is called the *multiple shooting method* [2, Ch. 4.4.3]. For this, the time interval $[0,T]$ is partitioned by shooting points $0 = t_{1} < t _{2} < \cdots< t_{N+1} =T$. On each subinterval $[t_{i}, t_{i+1}]$, we may compute a solution $y_{i}(t) = Y_{i}(t)c_{i} + w_{i}(t)$ similarly as before. The coefficient vectors $c_{i}\in\mathbb{R}^{s}$ are given by a linear system that contains the boundary and the continuity conditions in-between the time steps.

##### Remark 12

For the other cases, i.e., for $r>0$ (with holonomic constraints) or different values of $\nu$, we may need to use different techniques, depending in the structure of the BVP. In the case $r=0$, $\nu=0$ (cf. Sect. 3.5.1, where we obtain as an optimality system an index-1 DAE) and need to consider shooting methods. For this, we refer to [24]. In the case $r>0$, for which we obtain index-3 systems, we refer to [11] or, after an index reduction to index 2, also to [13].

### Riccati approach

In the linear case and if $\nu=0$, i.e., if no derivatives of $u$ appear in the optimality system (9a)–(9e), the BVP can be solved via a Riccati decoupling. This requires the formulation as a first-order system as in (13), which already is in the standard form considered; see, e.g., in [25, Ch. 5]. In the case of holonomic constraints, we can use the results on constrained Riccati equations given in [15], which readily apply to constrained multibody equations in the *Gear–Gupta–Leimkuhler* formulation [14].

## Numerical examples

In this section, we provide several numerical experiments. First, we consider the two-car system from Example 1, i.e., an example without holonomic constraints. Second, we add a third car, which then gives an index-7 DAE in the original formulation. Finally, we consider an overhead crane as an example with $r>0$, i.e., with a holonomic constraint. The code is written in *Python* and can be obtained from the author’s public *Github* repository [16].

### Two-car example

We consider the two-car example from the introduction; see Fig. 1. Recall that the equations of motion (1a)–(1c) form a DAE of index 5. As in [5, Ex. 3], the following parameters were used within the computations:

$$\begin{aligned} m_{1} = 2~\mbox{kg},\qquad m_{2} = 1~\mbox{kg}, \qquad k = 1~\tfrac{\mathrm{N}}{\mathrm{m}}, \qquad d = 0.5~\mbox{m}. \end{aligned}$$

#### Comparison of DAE and optimal control solution

The initial values within the computations are given by

$$\begin{aligned} x^{0}_{1} = 0.5~\mbox{m},\qquad v^{0}_{1} = 0~\tfrac{\mbox{m}}{\mbox{s}}, \qquad x^{0}_{2} = 0~\mbox{m}, \qquad v^{0}_{2}=0~\tfrac{\mbox{m}}{\mbox{s}}. \end{aligned}$$

For the definition of a rest-to-rest maneuver, we introduce the polynomial

$$\begin{aligned} p(s) = 1716 s^{7} - 9009s^{8} + 20020s^{9} - 24024s^{10 }+ 16380s^{11 }- 6006s^{12 }+ 924s^{13}. \end{aligned}$$

With this and $y_{0} = 0.5~\mbox{m}$, $y_{\text{f}}= 2.5~\mbox{m}$, we define on the time interval $[0, 4s]$ the target trajectory

29 $$\begin{aligned} y(t) = \textstyle\begin{cases} y_{0} & \text{if }0\leq t < 1, \\ y_{0} + p (\frac{t-1}{2} ) (y_{\text{f}}-y_{0}) & \text{if }1 \leq t \leq3, \\ y_{\text{f}} & \text{if } 3 < t \leq4. \end{cases}\displaystyle \end{aligned}$$

Note that $y$ is smooth enough, so that the DAE solution (which we refer to as an exact solution) exists. Recall that this requires consistent initial positions and initial velocities of the cars as mentioned in Remark 1, namely

$$\begin{aligned} y(0) = x^{0}_{1}, \qquad\dot{y}(0) = v^{0}_{1}, \qquad m_{1}\ddot{y}(0) = -\bigl(x^{0}_{1}-x^{0}_{2} - d\bigr), \qquad m_{1}y^{(3)}(0) = -\bigl(v^{0}_{1}-v ^{0}_{2}\bigr). \end{aligned}$$

We compare the exact solutions, which are readily computable from the systems equation, to the trajectories and input forces obtained from solving the associated optimal control problem of Configuration 3 with parameters $Q=S=1$ and for varying $R_{0}= \beta\in\mathbb{R}$. The occurring linear boundary value problem is solved by finite differences on a regular grid of size $\tau= 0.01~\mbox{s}$.

**Fig. 2:**
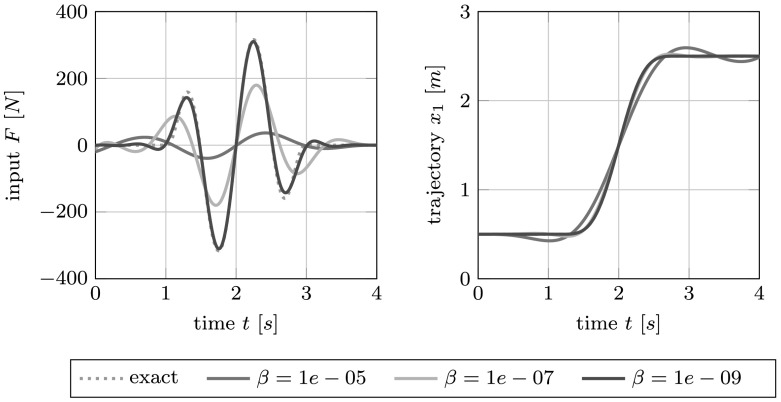
The exact input force $F$ (DAE solution) and the forces obtained through the optimal control formulation for different values of the penalization parameter $\beta$ (*left*) and the corresponding trajectories (*right*) for the two-car example

As expected, depending on the penalization parameter $\beta$, the optimal control approach leads to input forces that are smaller than the exact force $F$; see Fig. 2 (left). The reduction of the amplitude is best seen for large values of $\beta$. The optimal control problem is a compromise of costs and accuracy, as can be seen from the deviations from the target trajectory, which decrease for smaller values of the penalization parameter $\beta$; see Fig. 2 (right).

#### Feedback representations of the optimization solutions

Another advantage of the optimization approach is that the optimal control can be realized as a feedback. In fact, the first-order optimality conditions (9a)–(9e) suggest that $u$ depends linearly on $\lambda$, which depends, possibly nonlinearly, on the state $x$. For the considered linear case of Example 1, the optimality system can be solved via a differential Riccati equation [25, Ch. 5.1], which directly leads to a feedback representation of the optimal control.

**Fig. 3:**
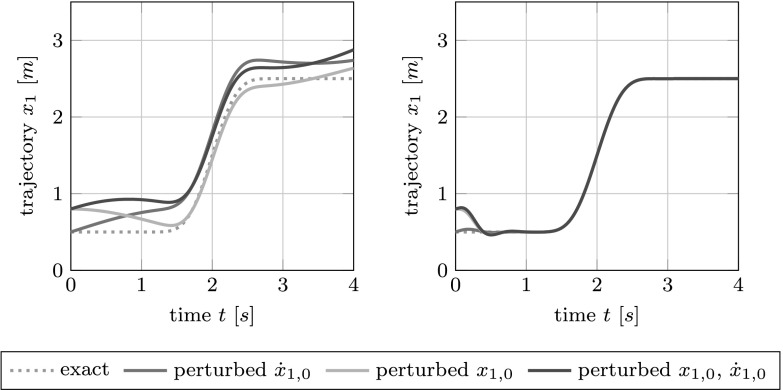
Benefits of the feedback representation: The output obtained from applying the exact control solution directly (*left*) and the output obtained via a feedback representation of the control solution of the optimal control problem (*right*) in the case of perturbed initial values

We stay with the example of the 2-car setup to illustrate the benefits of the feedback representation. If we simply apply the known exact control solution to the considered system, then a perturbation, e.g., in the initial position or initial velocity, necessarily leads to a drift off the desired trajectory; 0 see Fig. 3 (left). In contrast, the feedback solution of the optimal control problem with $\beta_{0}=10^{-9}$ detects and damps possible perturbations; see Fig. 3 (right).

### Three-car example

In this subsection, we add an additional car, i.e., we consider Example 2 with $n=3$. This means that the positions of the bodies are given by $x_{1}$, $x_{2}$, and $x_{3}$, where the trajectory of $x_{1}$ is prescribed by $y$. Recall that this gives a DAE of index 7 rather than of index 5 as in the previous example. As parameters, we set

$$\begin{aligned} m_{1} = m_{2} = 1~\mbox{kg}, \qquad m_{3} = 2~\mbox{kg}, \qquad k = 1~\tfrac{\mbox{N}}{\mbox{m}}, \qquad d = 0.5~\mbox{m}. \end{aligned}$$

As initial conditions we have

$$\begin{aligned} x^{0}_{1} = 0.5~\mbox{m},\ v^{0}_{1} = 0~\tfrac{\mbox{m}}{\mbox{s}}, \qquad x^{0}_{2} = 0~\mbox{m},\ v^{0}_{2} = 0~\tfrac{\mbox{m}}{\mbox{s}}, \qquad x^{0}_{3} = -0.5~\mbox{m},\ v^{0}_{3} = 0\,\tfrac{\mbox{m}}{\mbox{s}}. \end{aligned}$$

For the simulation, we consider the same rest-to-rest maneuver as in (29). As required, the prescribed trajectory is six times continuously differentiable, and the initial conditions satisfy all consistency conditions; cf. Assumption 1 and Remark 1.

**Fig. 4:**
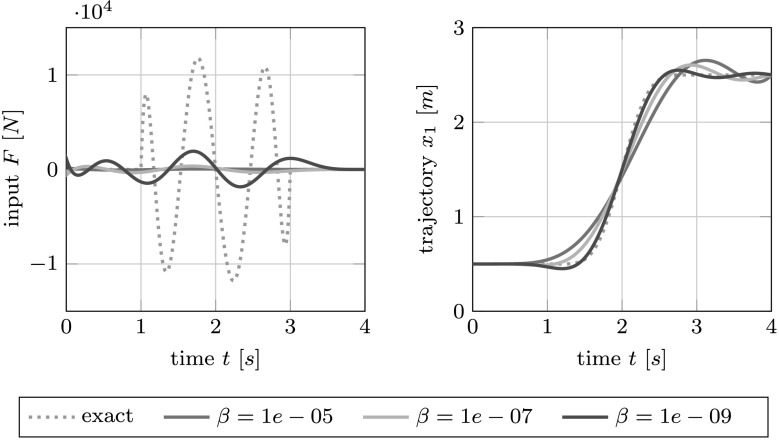
The exact input force $F$ (DAE solution) and the forces obtained through the optimal control formulation for different values of the penalization parameter $\beta$ (*left*) and the corresponding trajectories (*right*) for the three-car example

The exact solution and the results from the optimal control problem for different values of the penalization parameter are shown in Fig. 4. The weak coupling from the input on the third car to the output measured on the first car is apparent in the large peaks in the exact input force. The optimization approach leads to significantly reduced amplitudes in the input at the expense of a certain deviation from the prescribed target trajectory.

### Overhead crane

**Fig. 5:**
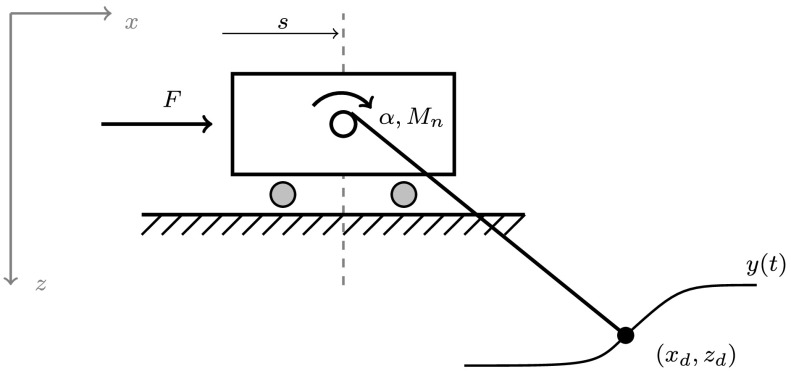
Overhead trolley crane with the notation of the rotationless formulation introduced in [10]

The servo-constraint problem of this section was originally formulated in terms of minimal coordinates in [5] and was recast in redundant coordinates in [10]; see Fig. 5. We follow here the latter approach, which then fits into the framework of Sect. 2.

For this, we consider the state and input variables

$$\begin{aligned} x = [s,\ \alpha,\ x_{d},\ z_{d}]^{{\mathsf{T}}}, \qquad u = [F,\ M _{n}]^{{\mathsf{T}}}. \end{aligned}$$

With these redundant coordinates (one additional variable and one additional equation), we need to add one holonomic constraint and the corresponding Lagrange multiplier $p \in\mathbb{R}$. The overall system reads

30a $$\begin{aligned} \left[ \textstyle\begin{array}{c@{\quad}c@{\quad}c@{\quad}c} m_{t}& & & \\ & J & & \\ & & m & \\ & & & m \end{array}\displaystyle \right] \begin{bmatrix} \ddot{s}\\ \ddot{\alpha}\\ \ddot{x}_{d}\\ \ddot{z}_{d} \end{bmatrix} - G^{{\mathsf{T}}}(x) p - \left[ \textstyle\begin{array}{c@{\quad}c} 1 & 0 \\ 0 & 1 \\ 0 & 0\\ 0 & 0 \end{array}\displaystyle \right] u &= \begin{bmatrix} 0\\ -rmg\\ 0\\ mg \end{bmatrix} , \end{aligned}$$

30b $$\begin{aligned} (x_{d}-s)^{2} + z_{d}^{2} - (r \alpha)^{2} &= 0, \end{aligned}$$

30c $$\begin{aligned} \begin{bmatrix} x_{d}\\ z_{d} \end{bmatrix} - y &= 0 \end{aligned}$$

with

$$\begin{aligned} G(x) = 2 \left[ \textstyle\begin{array}{c@{\quad}c@{\quad}c@{\quad}c} s-x_{d} & -r^{2}\alpha & x_{d} -s & z_{d} \end{array}\displaystyle \right] , \qquad \bigl(G(x)^{{\mathsf{T}}}p\bigr)_{x} = 2p \left[ \textstyle\begin{array}{c@{\quad}c@{\quad}c@{\quad}c} 1 & 0 & -1 & 0 \\ 0 & -r^{2} & 0 & 0 \\ -1 & 0 & 1 & 0 \\ 0 & 0 & 0 & 1 \end{array}\displaystyle \right] . \end{aligned}$$

In the optimal control approach, with a cost functional as in Configuration 3, the solution is obtained through the solution of the additional adjoint equations (9c) and (9d), the gradient condition (9e), and the boundary conditions (10) and (11).

We consider the system parameters

$$\begin{aligned} m_{t} = 10~\mbox{kg},\qquad J = 0.1~\mbox{Nm}, \qquad m = 1~\mbox{kg}, \qquad r = 0.1~\mbox{m}, \qquad g = 9.81~\tfrac{\mbox{m}}{\mbox{s}^{2}} \end{aligned}$$

with initial values

$$\begin{aligned} x_{0} = [0~\mbox{m},\ 40~\mbox{m}, \ 0~\mbox{m},\ 4~\mbox{m}]^{{\mathsf{T}}} \quad\text{and}\quad \dot{x}_{0} = [0,\ 0, \ 0,\ 0]^{{\mathsf{T}}}. \end{aligned}$$

Furthermore, we consider a target trajectory as defined in (29) but on the time interval $[0, 6\, \mathrm{s}]$ and with the vector-valued starting and terminal points

$$\begin{aligned} y_{0} = [0,\ 4]^{{\mathsf{T}}}\quad\text{and}\quad y_{f} = [1,\ 5]^{{\mathsf{T}}}. \end{aligned}$$

We linearize the resulting nonlinear boundary value problems with holonomic constraints around the constant solution obtained with $u\equiv[0,\ 0]^{{\mathsf{T}}}$ and solve it via finite differences. The computed approximation to the optimal control is then evaluated in the actual nonlinear model (30a), (30b) and compared to the analytical solution of (30a)–(30c).

**Fig. 6:**
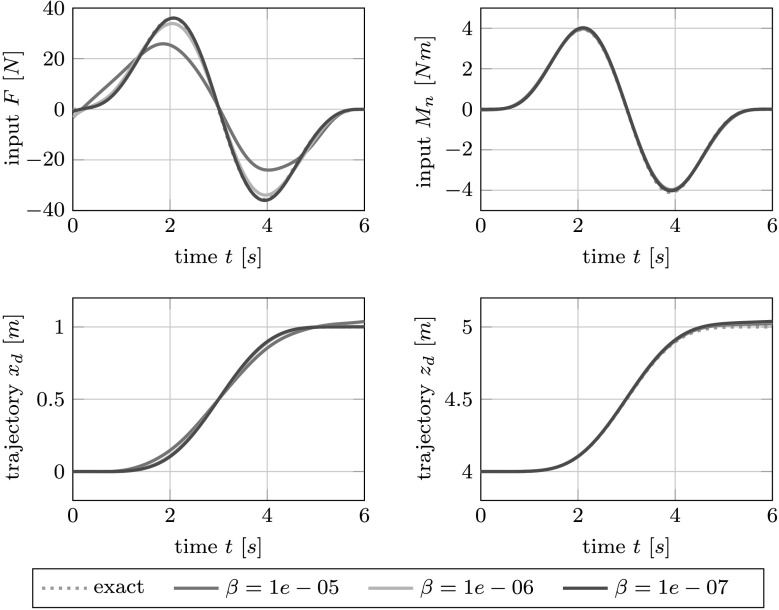
The forces $F$ and $M_{n}$ obtained through linearization and optimization for varying penalization parameter $\beta$ (*top*) and the resulting trajectories (*bottom*) for the example of the overhead crane

As the plots in Fig. 6 show, this combined linearization and optimization approach leads to a decent approximation of the actual control.

## Conclusion

Within this paper, we have considered mechanical systems with a partly specified motion, which are usually modeled by DAEs of index $\ge5$. Such models require strong regularity assumptions, and their numerical treatment is extremely challenging because of the sensitivity to perturbations. Because of this, an alternative modeling approach was introduced, which relaxes the prescribed servo constraint and considers a minimization problem instead. By this we decrease the possible errors that occur in the simulation of a high-index DAE but include an additional error since we do not satisfy the constraint exactly. However, this modeling error is controllable by the penalization parameters.

By numerical examples we have shown the advantages of the optimal control approach. First, the resulting control effort is much smaller at the price of only a small error in the constraint and, thus, more realistic since this corresponds to a reduction of costs in real-world applications. Second, the approach is less sensitive to perturbations such as inconsistent initial data.

Finally, we point out that the usefulness of the proposed approach needs to be determined in experiments as well. Thus, the next steps in this development direction will be the inclusion of sensor and actuator dynamics and the implementation in an experimental setup.
